# Feasibility and Safety of Mark-Guided Submucosal Tunneling Endoscopic Resection for Treatment of Esophageal Submucosal Tumors Originating from the Muscularis Propria: A Single-Center Retrospective Study

**DOI:** 10.1155/2021/9916927

**Published:** 2021-06-30

**Authors:** Ben-hua Wu, Rui-yue Shi, Hai-yang Zhang, Ting-ting Liu, Yan-hui Tian, Feng Xiong, Zheng-lei Xu, Ding-guo Zhang, De-feng Li, Jun Yao, Li-sheng Wang

**Affiliations:** Department of Gastroenterology, Shenzhen People's Hospital, The Second Clinical Medical College, Jinan University, The First Affiliated Hospital, Southern University of Science and Technology, Shenzhen 518020, Guangdong, China

## Abstract

**Background:**

Submucosal tunneling endoscopic resection (STER) has effectively removed esophageal submucosal tumors (SMTs) originating from the muscularis propria (MP) layer. However, clinical failure and adverse events of STER remain concerned. In this study, we described a mark-guided STER (markings before creating entry point) and evaluated its feasibility and safety for esophageal SMTs originating from MP.

**Methods:**

Patients receiving the mark-guided STER from October 2017 to July 2020 were included and followed up (ranged from 3 to 30 months). The primary outcomes included complete resection, en bloc resection, and R0 resection rates. The secondary outcomes included procedure duration, main complication, and residual lesions.

**Results:**

A total of 242 patients with 242 SMTs (median diameter of 22 mm, ranging from 7 mm to 40 mm) received the mark-guided STER. The median procedure duration was 55 min (ranging from 35 min to 115 min). The complete resection, en bloc resection, and R0 resection rates were 100%, 98.3%, and 97.5%, respectively. The adverse event rate was 4.5%. However, there was no severe complication. No residual SMTs were detected during the follow-up period. Logistic regression demonstrated that the SMT size and procedure duration were independent factors associated with en bloc resection (*P*=0.02 and *P*=0.04, respectively). Moreover, logistic regression demonstrated that the SMT size was an independent risk factor for main complications (*P*=0.02).

**Conclusion:**

Mark-guided STER was feasible and safe to remove esophageal SMTs ≦40 mm. However, it is necessary to further verify the feasibility and safety for the esophageal SMTs >40 mm.

## 1. Introduction

Esophageal submucosal tumors (SMTs) are unexpectedly discovered during upper gastrointestinal endoscopy, which typically appear as tissue protuberance with intact mucosa, and its estimated overall prevalence is less than 1% of all esophageal tumors [1]. Esophageal SMTs commonly consist of leiomyomas and gastrointestinal stromal tumors (GISTs), while most of them are usually benign and free of clinical symptoms [2]. However, some of SMTs, especially those originating from the muscularis propria (MP) layer or those with large size, do have malignant potential [3–5].

The management of esophageal SMTs remains controversial. The American Gastroenterological Association (AGA) recommends that GISTs >3 cm should be removed, whereas those ≦3 cm should be followed up [6]. However, the National Comprehensive Cancer Network (NCCN) guideline recommends that GISTs >2 cm should be removed, whereas those ≦2 cm should be carefully monitored [2]. It would be best to obtain a pathological diagnosis of esophageal SMTs in order to guide the next step; however, it seems to be difficult [7, 8]. Besides, long-time surveillance might impose a tremendous emotional burden and increase the financial burden on patients, leading to the development of malignancy [9]. Therefore, early intervention of esophageal SMTs may be necessary.

Several techniques, including endoscopic submucosal dissection (ESD), endoscopic submucosal enucleation (ESE), endoscopic full-thickness resection (EFR), thoracoscopic enucleation, and submucosal tunneling endoscopic resection (STER), have been proved to be effective for esophageal SMTs [10–13]. However, STER is gradually recommended because it possesses more advantages than ESD, ESE, EFR, and thoracoscopic enucleation [14–16]. In 2012, inspired by peroral endoscopic myotomy (POEM), STER was firstly developed to remove esophageal SMTs originating from the MP layer [17]. Nevertheless, STER is a difficult and experience-requiring technique because a relatively straight tunnel is the key to successfully remove SMTs during procedure [18]. In the present study, we described a mark-guided STER and evaluated its feasibility and safety for the treatment of SMTs originating from the MP layer.

## 2. Methods

### 2.1. Patients

In this single-center retrospective study, 242 consecutive patients including 242 esophageal SETs, who underwent STER, were initially extracted between October 2017 and July 2020. Written informed consent was obtained from each participant before STER. The study was conducted in accordance with the guidelines of the Declaration of Helsinki and approved by the Ethics Committee of Shenzhen People's Hospital.

Patients who met the following criteria were included: (1) diagnosis of esophageal SMTs originating from MP layer was confirmed by endoscopic ultrasonography (EUS) and computed tomography (CT); (2) sign of endophytic and intraluminal SMTs without ulceration; (3) no evidence of malignancy or metastasis or invasion outside the esophageal tract; (4) age ≥18 years old; (5) SMT sizes ≦40 mm; (6) blood cell count and prothrombin time within normal level; (7) patients taking antithrombotic agents needed to withhold 1 week or replace heparin before STER; and (8) signed informed consent. Exclusion criteria were set as follows: (1) reluctance to undergo STER; (2) inability to sign informed consent; (3) inability to tolerate anesthesia; (4) patients with severe cardiorespiratory dysfunction; (5) high-risk operation or pregnancy; (6) patients with multiple esophageal SMTs; and (7) patients who were lost during follow-up.

### 2.2. Mark-Guided STER Procedure

**Figure 1:**
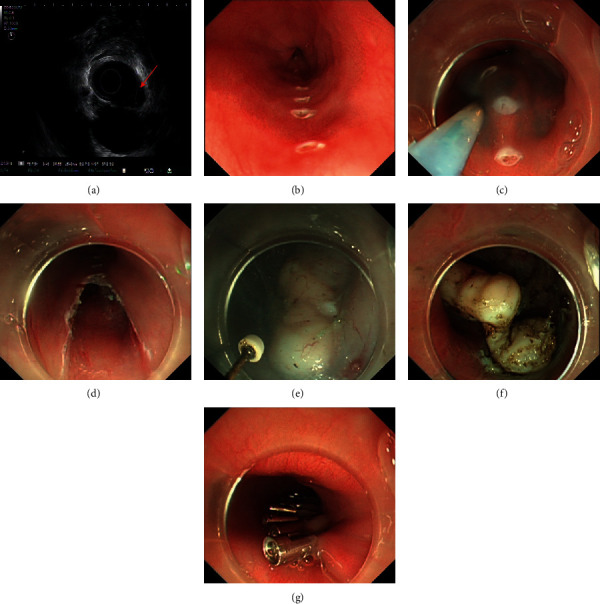
The mark-guided STER procedure. (a) EUS confirmed the characteristics of esophageal SMT. (b) Marking from esophageal mucosal surface of SMTs to 5.0 cm to the proximal margin of the SMT. (c) Injecting diluted indigo carmine through the marks. (d) Creating entry point through longitudinal incision. (e) Creating a longitudinal tunnel. (f) Dissecting the SMT. (g) Closing the entry point.

Mark-guided STER was performed mainly according to a previously established procedure with some modifications [17]. The patients were placed in a left lateral decubitus position (LLDP) under general anesthesia with endotracheal intubation. A carbon dioxide (CO_2_) insufflator was used during the procedure. The key steps were as follows: (1) the characteristics of esophageal SMTs, such as location, size, and depth, were assessed using EUS before the procedure (Figure 1(a)). (2) The region from esophageal mucosal surface of SMTs to 5.0 cm to the proximal margin of the SMTs was marked using dual knife (Figure 1(b)). (3) Diluted indigo carmine was injected into a fluid cushion through the marks (Figure 1(c)). (4) A longitudinal incision of about 2 cm was made using dual knife to create entry point (Figure 1(d)). (5) A longitudinal tunnel was created between the mucosal and muscular layers and terminated at about 2 cm distal to the SMTs (Figure 1(e)). A satisfactory endoscopic view of the SMTs and sufficient space were made to dissect the SMTs. (6) The SMTs were carefully dissected using insulation-tip knife and retrieved from the tunnel entry (Figure 1(f)). (7) The tunnel entry was closed after hot biopsy forceps was used for hemostasis (Figure 1(g)).

### 2.3. Perioperative Management

All patients were hospitalized and fasted for 8 h before the procedure. Antibiotics were routinely administered to prevent the infection for 3 days. Meanwhile, all patients were intravenously administered with prophylactic proton pump inhibitors (PPIs) (esomeprazole, 40 mg, twice daily) for 3 days, after which oral PPIs (esomeprazole, 20 mg, twice daily) were prescribed for 8 weeks after the procedure. If patients showed no evidence of complications for 3 days, a full fluid diet and normal food were gradually resumed in the next 2 weeks.

Possible complications were monitored, such as postprocedure bleeding, pneumothorax or pleural effusion, esophageal-pleural fistula, pulmonary infection, severe chest pain, and perforation.

### 2.4. Pathology Evaluation

After excision, the specimens were fixed in 10% buffered formalin, embedded with paraffin, and sectioned for pathological examination by pathologists. Immunohistochemical staining was used to determine undefined pathological type.

### 2.5. Follow-Up

Surveillance endoscopy was performed to assess the wound healing and monitor the residual and recurrent lesions at 3, 6, and 12 months and then once yearly thereafter. For patients diagnosed with GISTs, a contrast-enhanced CT was recommended at every 12 months.

### 2.6. Outcomes

The primary outcomes included mark-guided STER-related complete resection, en bloc resection, and R0 resection rates. The complete resection was regarded as no residual lesion fragment on endoscopic views at the resection site. En bloc resection was regarded as completion resection of the SMTs with single piece. R0 resection was regarded as en bloc resection with laterally and basically free pathological margin.

The second outcomes included procedure duration, main complications, residual or recurrent lesions, hospital stay, and hospitalization expenditure. Procedure duration was determined from the submucosal injection to the closure of the tunnel entry point. Main complications included intraprocedure or postprocedure bleeding, pneumothorax or pleural effusion, esophageal-pleural fistula, pulmonary infection, severe chest pain, and perforation. Residual lesion was regarded as the SMTs detected at the original site within 6 months during follow-up, whereas recurrent lesion was regarded as the SMTs detected at the original site more than 6 months during follow-up [19].

### 2.7. Statistical Analysis

Continuous variables were expressed as mean ± standard deviation (SD) or median (interquartile range, IQR, 25%–75%). Categorical variables were shown as proportions. Logistic regression was performed to assess possible factors associated with en bloc resection and main complications. All analyses were performed using the SPSS 23.0 software package (SPSS Company, Chicago, IL, USA). *P* values <0.05 were considered statistically significant.

## 3. Results

### 3.1. Clinical Characteristics

From October 2017 to July 2020, a total of 242 consecutive patients with 242 esophageal SMTs received mark-guided STER in our clinical center. The median age was 54 years (ranging from 30 to 75 years) with a male/female ratio of 131/111. Of these 242 patients, 96 patients (38.0%) had typical symptoms, such as dysphagia and choking, whereas 146 patients (62.0%) had atypical symptoms, such as regurgitation and epigastric discomfort. Of these 242 SMTs, 42 (17.4%) were located in the upper esophagus, 105 (43.3%) were found in the middle esophagus, and 95 (39.3%) were detected in the lower esophagus. The median diameter of the SMTs was 22 mm (ranging from 7 mm to 40 mm). The postprocedure samples showed that 220 SMTs (90.9%) had regular shapes, and 22 SMTs (9.1%) had irregular shapes. The postprocedure histology revealed 235 leiomyomas (97.1%), five GISTs (2.1%), and two schwannomas (0.8%). Moreover, all of these five GISTs were classified to be very risk.  Table 1 summarizes the detailed characteristics.

### 3.2. Effectiveness of Mark-Guided STER

The mark-guided STER was performed in all of 242 esophageal SMTs. Complete resection was achieved in 242 SMTs (100%). En bloc resection was achieved in 238 SMTs (98.3%). R0 resection was achieved in 236 SMTs (97.5%). The median procedure duration was 55 min (ranging from 35 min to 115 min) (Table 2).

### 3.3. Safety of Mark-Guided STER

A total of 12 patients (4.5%) developed main adverse events. There were five cases of intraprocedure bleeding (2.1%), two cases of pulmonary infection (0.8%), four cases of severe chest pain (1.7%), and one case of intraprocedure perforation (0.4%) (Table 2). No postprocedure bleeding, pleural fistula, esophageal-pleural effusion, and postprocedure perforation occurred during or after the procedure. Hot biopsy forceps successfully stopped the bleeding for five cases of intraprocedure bleeding without blood transfusion or surgery intervention or angiography intervention. Moreover, other patients with complications recovered smoothly after the conservative treatment.

### 3.4. Follow-Up

All patients received follow-up, and the median period was 17 months (ranging from 3 to 30 months). Moreover, no residual and recurrent SMTs were detected in any patients during the follow-up. Although five cases of leiomyomas and one case of GISTs did not achieve R0 resection, no recurrent tissues were detected at the original site (Table 2).

### 3.5. Factors Associated with En Bloc Resection

Univariate logistic regression showed that the SMT size, SMT shape, SMT pathology, and procedure duration were associated with R0 resection (*P*=0.006, *P*=0.03, *P*=0.02, and *P*=0.001, respectively), while gender, age, and SMT location were not associated with R0 resection (*P*=0.99, *P*=0.87, and *P*=0.54, respectively) (Table 3). However, multivariate logistic regression demonstrated that SMT size and procedure duration were independent factors associated with en bloc resection (*P*=0.02 and *P*=0.04, respectively) (Table 3).

### 3.6. Factors Associated with Main Complications

Univariate logistic regression showed that the SMT size, SMT shape, and procedure duration were associated with main complications (*P* < 0.001, *P* < 0.001, and *P* < 0.001, respectively), while gender, age, SMs location, and SMT pathology were not associated with main complications (*P*=0.56, *P*=0.67, *P*=0.31, and *P*=0.80, respectively) (Table 4). However, multivariate logistic regression demonstrated that SMT size was an independent risk factor for main complications (*P*=0.02) (Table 4).

## 4. Discussion

To the best of our knowledge, we, for the first time, described mark-guided STER for the treatment of esophageal SMTs, and the feasibility and safety of such technique were also evaluated. Our results indicated that the complete resection rate was 100%, whereas the en bloc resection rate and R0 resection rate were 98.3% and 97.5%, respectively. Moreover, the overall adverse event rate was 4.5%. However, there was no severe complication. Indeed, there were no residual or recurrent SMTs during the follow-up period. Multivariate logistic regression demonstrated that SMT size and procedure duration were independent factors associated with en bloc resection. Furthermore, multivariate logistic regression demonstrated that SMT size was an independent risk factor for main complications. Therefore, mark-guided STER was a feasible and safety modality for the treatment of esophageal SMTs originating from the MP layer.

Du et al. have reported that STER is proved to be effective and safe for the treatment of esophageal SMTs originating from MP layers [19]. Although the complete resection rate and residual or recurrent rate are comparable between abovementioned study and our study, their en bloc resection rate is dramatically less than our results (78.7% vs. 98.3%). The reason may be attributed due to the fact that the size of SMTs in abovementioned study is more lager than that of our study (60 mm vs. 40 mm, respectively). Furthermore, we demonstrated that the size of SMTs was an independent factor associated with en bloc resection. Indeed, Chai et al. have shown that the STER-related en bloc resection rate is 100% for the treatment of esophageal SMTs ˂20 mm, while the en bloc resection rate is decreased to 71.4% when the esophageal SMTs are ≥20 mm [20].

Chen et al. have reported that the adverse event rate of STER is 13.3% for the treatment of esophageal SMTs (ranging 10 mm to 50 mm), which is higher than our results (13.3% vs. 4.5%, respectively) [14]. This discrepancy might be attributed to the small size of esophageal SMTs in our study. Meanwhile, Chen et al. have revealed that the adverse event rate of STER when treating SMTs ˂2 cm is dramatically lower compared with SMTs ≥2 cm (4.3% vs. 25.6%, respectively) [14]. Moreover, we demonstrated that the size of SMTs was an independent risk factor for main complications during the STER procedure.

Wang et al. have evaluated the feasibility and safety of STER for the treatment of large esophageal SMTs (ranging from 30 mm to 70 mm) originating from the MP layer and demonstrated that complete resection, en bloc resection, and adverse events rates are 100%, 85.2%, and 14.8%, respectively [21]. Chen et al. have shown that the en bloc resection and adverse event rates are 84.6% and 7.7%, respectively, when large esophageal SMTs (˃50 mm) originating from the MP layer are removed by STER [22]. In this study, our data revealed that mark-guided STER not only achieved a higher en bloc resection rate (98.3%) but also yielded a lower adverse event rate (4.5%). However, all of the esophageal SMTs receiving mark-guided STER were less than 40 mm. Therefore, the feasibility and safety of the mark-guided STER for the treatment of large esophageal SMTs should be further confirmed.

In this study, we found that the procedure duration of mark-guided STER was shorter compared with several previous studies, whereas the en bloc resection rate of mark-guided STER was prominently higher than that of these studies [15, 16, 21]. However, multivariate logistic regression demonstrated that procedure duration was an independent factor associated with en bloc resection. It seemed that we drew a contradictory conclusion in this study. There were two possible reasons. One could be that the size of esophageal SMTs was smaller in our study. The other one might be that the mark-guided technique could reduce procedure duration and improve the endoscopic vision.

There were several strengths mentioned in this study. First, the mark-guided STER could create a straight tunnel during procedure, which could easily find the esophageal SMTs and decrease the procedure duration. Second, the mark-guided STER could create large submucosal tunnel lumen through mark-guided sufficient submucosal injection, which could improve the operative vision, increase the en bloc resection rate, and decrease the main complications. However, this study has some limitations. First, it was designed as a single-center, retrospective study. Second, the endoscopists involved in the study were experienced in POEM. Therefore, we could not guarantee whether our results were generally reproducible. Third, the feasibility and safety of mark-guided STER were compared with literature using traditional STER. Therefore, a prospective randomized controlled trial will be performed to further assess the feasibility and safety and compare the mark-guided STER with the traditional STER. Finally, the follow-up time was quite short in this study.

Collectively, the mark-guided STER was feasible and safe for the treatment of esophageal SMTs ≦40 mm originating from the MP layer. However, it is necessary to further verify the feasibility and safety of mark-guided STER for the treatment of esophageal SMTs ˃40 mm originating from the MP layer.

## Figures and Tables

**Table 1:** Baseline characteristics.

Characteristics	
*Gender (n, %)*	
Male	131 (54.1%)
Female	111 (45.9%)
Age (years)	54 (30–75)

*Symptoms (n, %)*	
Typical	96 (39.7%)
Atypical	146 (60.3%)

*Location (n, %)*	
Upper	42 (17.4%)
Middle	105 (43.3%)
Lower	95 (39.3%)
Size (mm)	22 (7–40)

*Shape (n, %)*	
Regular	220 (90.9%)
Irregular	22 (9.1%)

*Histology (n, %)*	
Leiomyomas	235 (97.1%)
GISTs	5 (2.1%)
Schwannoma	2 (0.8%)

Note: GISTs, gastrointestinal stromal tumors.

**Table 2:** The feasibility and safety of the mark-guided STER.

Outcomes	
Complete resection (*n*, %)	242 (100%)
En bloc resection (*n*, %)	238 (98.3%)
R0 resection (*n*, %)	236 (97.5%)
Procedure duration (min)	55 (35–135)
Main complication (*n*, %)	12 (4.5%)
Intraprocedure bleeding (*n*, %)	5 (2.1%)
Pulmonary infection (*n*, %)	2(0.8%)
Severe chest pain (*n*, %)	4 (1.7%)
Intraprocedure perforation (*n*, %)	1 (0.4%)
Postprocedure bleeding (*n*, %)	0
Pleural fistula (*n*, %)	0
Esophageal-pleural effusion (*n*, %)	0
Postprocedure perforation (*n*, %)	0
Follow-up (months)	17 (3–30)
Residual (*n*, %)	0
Recurrence (*n*, %)	0

**Table 3:** Logistic regression analysis associated the factors with en bloc resection.

	Univariate			Multivariate		
	OR	95% CI	*P* value	OR	95% CI	*P* value
Gender						
Female	Reference			Reference		
Male	0	0	0.99	0	0	0.99
Age	1.01	0.92–1.11	0.87	1.003	0.74–1.36	0.98
SMTs location	1.52	0.40–5.78	0.54	1.78	0.40–8.12	0.76
SMTs size	0.11	0.01–0.29	0.006	0.03	0.01–0.45	0.02
SMTs shapes	0.11	0.01–0.80	0.03	0.41	0.20–1.10	0.35
SMTs pathology	1.94	1.65–22.81	0.02	6.32	2.78–24.23	0.25
Procedure duration	0.90	0.85–0.96	0.001	0.91	0.86–0.97	0.04

Note: OR, odds ratio; CI, confidence interval; SMTs, submucosal tumors.

**Table 4:** Logistic regression analysis associated risk factors with main complications.

	Univariate			Multivariate		
	OR	95% CI	*P* value	OR	95% CI	*P* value
Gender						
Female	Reference			Reference		
Male	1.44	0.43–4.85	0.56	0.74	0.11–4.46	0.72
Age	0.99	0.93–1.05	0.67	0.98	0.63–1.52	0.92
SMTs location	0.65	0.29–1.48	0.31	1.49	0.32–5.68	0.93
SMTs size	23.56	16.76–22.10	<0.001	24.31	17.48–24.39	0.02
SMTs shapes	9.89	2.74–35.49	<0.001	11.65	3.52–37.05	0.07
SMTs pathology	1.62	0.04–69.11	0.80	1.47	0.09–23.81	0.78
Procedure duration	1.15	1.09–1.23	<0.001	0.74	0.53–1.05	0.09

Note: OR odds ratio; CI, confidence interval; SMTs, submucosal tumors.

## Data Availability

All data generated or analyzed during this study are available from the corresponding author upon reasonable request.
